# From History to Plan: A Comprehensive Audit of Admission Documentation Practices in the Pulmonology Department at Medical Teaching Institute (MTI) Mardan Medical Complex, Pakistan

**DOI:** 10.7759/cureus.92860

**Published:** 2025-09-21

**Authors:** Sajjad Ali, Sana Ullah, Saima Aslam, Salim Muhammad, Miraj Ahmad, Mohammad Asim

**Affiliations:** 1 Department of Pulmonology, Mardan Medical Complex, Mardan, PAK; 2 Department of Gynecology, Peshawar General Hospital, Peshawar, PAK; 3 Department of Physiology, Bacha Khan Medical College, Mardan, PAK; 4 Department of Pulmonology, Peshawar General Hospital, Peshawar, PAK

**Keywords:** admission documentation, clinical audit, documentation compliance, medical records, nhs standards, pulmonology research, quality improvement projects

## Abstract

Background

Accurate admission documentation is essential for safe and effective clinical care. It facilitates diagnosis, treatment planning, team communication, legal protection, and quality improvement. However, audits globally, including in low-resource settings, report major gaps in documentation quality, which may jeopardize patient outcomes and healthcare operations.

Objective

The objective of this study is to assess the quality and completeness of admission documentation in the Pulmonology Unit of MTI Mardan Medical Complex (MMC), Pakistan, against national and international standards.

Methods

This retrospective clinical audit reviewed 150 systematically selected patient admission records from January 1 to March 31, 2025. A structured audit tool, based on NHS and institutional guidelines, evaluated key documentation domains: demographics, history, examination, investigations, diagnosis, and initial management. Data were analyzed using descriptive statistics in Microsoft Excel.

Results

Only 1% (n = 1) of admission notes met all documentation standards. While demographics like name and age were documented in over 95% (n ≈ 143-150) of records, critical components were frequently missing. Presenting complaints were noted in 93% (n = 140), but detailed symptom analysis appeared in only 35% (n = 52), and red flag symptoms in 35% (n = 52). Medication history, allergy status, and vaccination history were missing in nearly all charts (98-100% missing, n = 147-150). Physical examination findings and vital signs were poorly recorded (3-61% = n = 5 to 92). Investigations and differential diagnoses were documented in less than 11% (n < 17) of cases. While 75% (n = 113) of records included an initial management plan, communication with patients or families was not documented in any case (0%, n = 0).

Conclusion

Significant documentation deficiencies were identified across multiple domains. Implementing a standardized admission template and conducting clinician training may improve compliance and patient care. Regular re-audits are recommended to assess ongoing progress.

## Introduction

Admission clerking, from patient history through examination and initial management plan, lays the foundation for inpatient care. High-quality documentation at admission is essential for safe and effective patient management. A complete record provides a baseline for diagnosis and treatment, facilitates communication among healthcare providers, and serves important legal, administrative, and educational purposes. Conversely, missing or inconsistent information in admission notes can lead to diagnostic errors, inappropriate treatments, and breakdowns in continuity of care. Documentation of patient care has often been described as the “Achilles’ heel” of clinical practice, with poor record-keeping linked to reduced care quality and compromised retrospective analyses [1].

Despite its importance, achieving comprehensive documentation remains challenging. Studies have shown that even in well-developed healthcare systems, admission records frequently omit key details. Smallwood et al. (2018) demonstrated that implementing structured admission proformas across multiple hospitals significantly improved the completeness of documentation, underlining the benefit of standardized forms [2]. In contrast, when clinicians use unstructured formats, critical information is more likely to be missed. Gaps in documentation can indirectly affect healthcare quality beyond immediate patient care; for instance, inaccurate or incomplete records impede data-driven decisions in funding, resource allocation, research, and quality improvement initiatives [3].

Significant documentation deficiencies are reported worldwide. A landmark multicenter study in the United States found that only 41.4% of recommended historical items were documented for adults with obstructive lung disease, indicating substantial variability and shortfalls in adherence to care standards [4]. Even with modern Electronic Health Records, important details are often underreported: one audit found that nearly 40% of major diagnoses were captured only in narrative free-text notes rather than in structured problem list fields, thereby limiting their accessibility for clinical decision support [5]. A national audit in Wales revealed incomplete admission note entries for acute asthma, and only about half of the cases had severity markers like peak expiratory flow and arterial blood gases documented on arrival [6]. Similarly, the EPOCONSUL audit of COPD outpatient clinics in Spain found that phenotype characteristics recommended by guidelines were documented in just 46.3% of patients, and formal risk stratification (per GOLD criteria) in only 21.9% [7]. These examples illustrate that documentation lapses are not confined to one region or setting.

In low- and middle-income countries, documentation problems can be even more pronounced. An audit of inpatient records at Basrah General Hospital in Iraq reported the overall documentation level to be “generally poor” in 78% of cases [1]. In that study, surgical departments showed particularly poor recording of patients’ histories and examination findings. A clinical audit in Sudan likewise found that crucial history elements were often missing - for example, only 41.1% of notes included the patient’s history of presenting complaint [8]. In Pakistan, documentation standards have also been suboptimal; a recent audit at a tertiary care dental hospital (using a CRABEL scoring system for record-keeping) showed that in 66% of cases there was no mention of the patient’s chief complaint, and nearly half lacked a documented diagnosis prior to treatment [9]. Taken together, these audits from various countries highlight pervasive inadequacies in medical record-keeping on admission. They reinforce the urgency for systematic improvements, such as structured documentation tools and clinician training, to ensure that patient records are complete and reliable.

Aim of the audit

In light of these concerns, we carried out a comprehensive audit of admission documentation practices in the Pulmonology Department at Medical Teaching Institute (MTI), Mardan Medical Complex, Pakistan. The aim was to evaluate current documentation against established standards and identify specific areas of non-compliance. By benchmarking our performance, we seek to inform targeted interventions (such as the introduction of standardized clerking proformas) to enhance the quality of admission records and ultimately improve patient care.

## Materials and methods

Setting and sample

This audit was conducted in the Pulmonology Ward of MTI Mardan Medical Complex (MMC), a tertiary care teaching hospital in Pakistan. The Pulmonology Ward was selected because this audit was initiated as a departmental quality improvement effort to assess and improve admission documentation within the Pulmonology Unit before scaling the initiative to other departments. We reviewed admission records over a three-month period from January 1 to March 31, 2025. During this interval, 750 patients were admitted to the Pulmonology Ward. Inclusion criteria were all adult patients (≥18 years) admitted to the Pulmonology Ward during the audit period with a documented physician admission note. Records were excluded if they were missing from the medical records office, were pediatric cases, or represented duplicate admissions for the same patient. Using a systematic sampling approach, we selected 150 admission cases (approximately 20% of admissions) for detailed review. Specifically, every 5th admission file from the chronological list was chosen to achieve a broad and unbiased sample across the entire audit period. The sample size was chosen in line with published guidance for retrospective clinical audits, which recommends reviewing 10-20% of total cases when full enumeration is impractical. No formal statistical formula was applied, as the objective was quality improvement rather than hypothesis testing. 

Audit criteria and standards

We employed a structured audit tool to assess each admission chart. The tool was developed by the authors based on the documentation guidelines outlined in the National Health Service (NHS) Health Records Standards. These standards are publicly available and considered open access. Prior to full-scale data collection, the tool was pilot tested on 10 randomly selected admission charts to assess clarity and inter-rater consistency. Minor revisions were made accordingly. All auditors were trained using the finalized tool to ensure consistency during the audit. The customized audit checklist includes domains such as demographics, presenting complaints, history of presenting illness, past and social history, medication and allergy history, physical examination, investigations, and initial diagnosis and plan. Since the final tool was tailored locally for the pulmonology context, the complete version is provided in Appendix A. It encompassed all critical components of a thorough admission clerking. The main domains and criteria evaluated were as follows:

Demographics and Initial Information

Documentation of patient’s name, age, gender, medical record (MR) number; an emergency contact number; the date and time of history-taking; and identification (name/designation) and signature of the admitting clinician. We also noted whether a preliminary assessment note by a postgraduate trainee (PG) on arrival was present. Standard: All these elements should be recorded for every admission (target compliance 95-100% for each sub-item).

Presenting Complaint

The primary symptom or reason for admission, ideally documented verbatim in the patient’s own words, along with the duration of that symptom. Standard: 100% of charts should record the presenting complaint and its duration.

History of Presenting Illness (HPI)

A narrative of the presenting complaint is presented with relevant details. This includes a description of the symptom(s), analysis of symptoms (onset, location, duration, frequency, exacerbating and relieving factors), any prior investigations or treatments related to the issue, the inclusion or exclusion of pertinent “red flag” symptoms (e.g. hemoptysis, fever, weight loss for a respiratory case), and an assessment of how the illness affects the patient’s daily activities or sleep (functional impact). Standard: Each of these aspects should be addressed in the history; target compliance for each sub-component is 90-100%.

Past Medical and Surgical History

Documentation of the patient’s previously diagnosed illnesses or chronic conditions (especially respiratory diseases like asthma or COPD), past hospitalizations or surgeries, and any relevant past health events. Standard: Should be recorded in all cases (target 95-100%).

Family History

Notation of any family history of pulmonary diseases (e.g. tuberculosis, asthma, lung cancer) or other hereditary conditions. Standard: Record if relevant; expected in nearly all charts given its potential importance (target ~95%).

Social History

This includes smoking history (current or past use of tobacco, with pack-years for smokers), exposure to biomass smoke or other environmental pollutants, any occupational dust or chemical exposures, alcohol consumption or illicit drug use, and living conditions (e.g. crowded home environment, which could increase tuberculosis risk). Standard: All relevant social factors should be queried and noted (target compliance ~90-100% for each, recognizing not all may apply to every patient).

Medication History and Allergies

A complete list of current medications (with doses if possible), any recent medications relevant to the presenting illness, and documentation of drug allergies or important immunizations (particularly influenza, pneumococcal, and COVID-19 vaccination status in a respiratory patient). Standard: 100% of charts should list current medications and allergies; vaccination status should be noted as applicable (targets ~90-100%).

Review of Systems (ROS)

A brief review of other major systems beyond the respiratory system - for instance, cardiovascular, gastrointestinal, genitourinary, neurologic - to identify any related symptoms. Particular attention to respiratory-associated systems (e.g. cardiovascular for cor pulmonale signs) is expected. Standard: A general ROS should be documented in all cases (target ~90% overall; 100% for the respiratory system).

Physical Examination

Documentation of vital signs (temperature, blood pressure, heart rate, respiratory rate, oxygen saturation), general examination (e.g. consciousness level, any respiratory distress signs such as use of accessory muscles or chest wall abnormalities), and a focused respiratory exam (inspection, palpation, percussion, auscultation findings). We also noted if a formal Early Warning Score (EWS/NEWS2) was calculated on admission, as per hospital protocol for acutely ill patients. Standard: Vital signs and a complete chest examination should be recorded for every patient (target 100% for vitals and chest auscultation, ~95% for other exam elements; an EWS should be documented whenever applicable).

Investigations

Initial diagnostic tests ordered and/or results obtained in the admission unit, such as chest X-ray (CXR), CT scan of chest, spirometry or peak flow readings, arterial blood gas (ABG), complete blood count (CBC) and other labs. Standard: Any investigations performed or ordered at admission should be noted in the chart (target ~90% of charts should list relevant investigations).

Impression and Plan

The admitting doctor’s initial impression or differential diagnosis based on the history and exam, and the initial management plan (including treatments started, further tests planned, consultations or referrals, and plan for monitoring). Also, whether the plan was communicated to the patient or family (an important aspect of informed consent and shared decision-making). Standard: Every chart should include an initial diagnosis or impression and a management plan (target ~95%); documentation of communication with patient/family about the plan is also expected as part of good practice (target 100%).

Consent and Confidentiality

We checked whether the notes indicated that patient consent was obtained for history/examination and that confidentiality was maintained (e.g. no unauthorized disclosure of patient information), as per professional standards. Standard: 100% compliance for recording consent and upholding confidentiality.

Each component was graded as “documented” (meets standard) or “not documented” (does not meet standard) in the chart. For elements with quantitative aspects (like vital signs), any omission of a key parameter was considered not meeting the standard. We tabulated the compliance for each item as a percentage of charts in which it was documented. The predetermined targets (drawn from NHS or institutional standards) were used to gauge adequacy. If a particular documentation element fell below its target compliance rate, it was flagged as an area needing improvement.

Data Collection and Analysis

Data were collected by three auditors (two medical officers and one senior resident) who were trained in using the audit tool for consistency. Each auditor reviewed a subset of files independently, after which the results were pooled. In cases of uncertainty in interpretation (e.g., unclear handwriting or ambiguous entries), the auditing team reached consensus through discussion.

The aggregated data were entered into Microsoft Excel for analysis. We calculated descriptive statistics, primarily the proportion of records complying with each documentation criterion. These proportions were compared against the target standards. The results are presented in both tabular and graphical formats to highlight the compliance rates and identify deficiencies at a glance. We constructed tables for each major documentation category (with rows for individual items and columns showing the target standard vs. the observed compliance percentage in our sample). Simple bar charts (figures) were generated to visualize the percentage of charts meeting each criterion, which aided in quickly conveying areas of high or low compliance.

No patient identifiers were recorded in the audit dataset, and the audit was conducted as part of an internal quality improvement initiative; thus, formal ethical approval was not required per institutional policy, though departmental permission was obtained. All audit procedures adhered to confidentiality standards, with patient charts accessed only by the auditing team in a secure records room.

## Results

A total of 150 patient admission charts were reviewed during the three-month audit period. Overall, the documentation quality was markedly substandard. Alarmingly, only 1 (1%) of the records fulfilled all the documentation standards assessed in the audit. This means that virtually no admission note contained a fully complete and structured history, examination, and management plan.

Demographics and initial clerking details

Basic demographic information was relatively well documented (Table 1). The patient’s name, age, and gender were recorded in 150 (100%) of the charts, while the medical record number was included in 146 (97%). Emergency contact numbers were noted in 140 (93%) of records. However, the date and time of history-taking were documented in only 42 (28%), and the name/designation of the admitting doctor appeared in just 17 (11%) of the records. A preliminary assessment note by a postgraduate trainee was found in 122 (81%) of the admission files. These compliance rates are summarized in Table 2.

**Table 1:** Demographic and Basic Admission Information (Pulmonology Ward, n = 150) MR: Medical Record; PG: Postgraduate Trainee. This table summarizes the compliance of admission records with essential demographic and initial clerking elements as per audit standards.

Parameter	Standard (%)	Observed (N (%))
Patient name, age, gender	100	150 (100%)
Emergency contact number	100	140 (93%)
Patient MR number	100	146 (97%)
Date and time of history-taking	95	42 (28%)
Name/designation of admitting doctor	100	17 (11%)
PG initial assessment note present	100	122 (81%)

**Table 2:** Compliance with Documentation Standards Across 13 Key Domains in Admission Notes (Pulmonology Ward, MTI Mardan Medical Complex, n = 150) This table summarizes the compliance of admission documentation across 13 critical domains, based on a retrospective audit of 150 admission records from the Pulmonology Ward at MTI Mardan Medical Complex. Compliance was defined as the presence of each documentation component in the admission note. Percentages reflect the proportion of records that met the documentation standard for each domain. The audit revealed high variability in compliance, with particularly low rates observed for medication history, investigations, and overall assessment. This highlights key areas for targeted improvement in clinical record-keeping.

S. No	Documentation Domain	Compliance n (%)
1	Patient Consent & Confidentiality	50 (23%)
2	Demographics	24 (16%)
3	Review of Systems	23 (15%)
4	Presenting Complaints	18 (12%)
5	History of Presenting Complaints	10 (7%)
6	Past Medical History	9 (6%)
7	Impression and Plan	7 (5%)
8	Family History	1 (1%)
9	Social History	1 (1%)
10	Physical Examination	0 (0%)
11	Investigations	0 (0%)
12	Medication History	5 (3%)
13	Overall Assessment	1 (1%)

The presenting complaint was mentioned in 140 (93%) of the charts, though only 112 (75%) of those captured the symptom in the patient’s own words. However, only 38 (25%) of records included a clear duration of the symptoms. A structured symptom analysis - including details like onset, location, frequency, and aggravating/relieving factors - was present in just 53 (35%) of charts. Documentation of prior investigations or treatments related to the current complaint was extremely limited, appearing in only six (4%) of cases. Red flag symptoms, such as hemoptysis or weight loss, were addressed in 53 (35%) of records, while functional impact (e.g., ability to perform daily activities or sleep disturbance) was recorded in 22 (15%) of charts.

Past medical, surgical, and family history

The patient’s past medical and surgical history was documented in 72 (48%) of cases. Family history was recorded in 30 (20%), but specific mention of familial pulmonary diseases such as tuberculosis or asthma was present in only a minority of these.

Social history

Smoking status was the most frequently documented social history item, appearing in 90 (60%) of admission notes. However, occupational exposure history was mentioned in only five (3%) of charts, and environmental exposures such as indoor smoke were documented in just two (1%) of records. Alcohol or illicit drug use was addressed in eight (5%), and housing conditions (e.g., overcrowding or poor ventilation) were noted in 15 (10%) of records.

Medication and allergy history

The most concerning finding was the complete absence of allergy documentation - 0 (0%) of charts recorded known drug allergies. Similarly, none of the charts (0 (0%)) included vaccination history, such as influenza, pneumococcal, or COVID-19 vaccines. Current medications were listed in only 6 (4%) of the files reviewed.

Review of systems (ROS)

A systems review beyond the respiratory system was documented in 86 (57%) of the admission notes. Cardiovascular symptoms were the most commonly included, while gastrointestinal, neurological, and genitourinary systems were often omitted. Given the respiratory context, a full review of related systems should have been included in nearly all records.

Physical examination

Lung auscultation findings were recorded in 92 (61%) of admission notes. However, documentation of vital signs - including temperature, pulse, respiratory rate, blood pressure, and oxygen saturation - was present in only 5 (3%) of charts. An Early Warning Score (EWS/NEWS2) was recorded in 45 (30%) of applicable cases. These findings highlight a significant oversight in initial clinical assessment documentation.

Investigations

Documentation of initial investigations, such as chest X-ray, complete blood count (CBC), ABG, or sputum studies, was present in 16 (11%) of the charts. The majority of records did not reference any diagnostic testing done at the time of admission, making it difficult to understand the clinical rationale or progression of care.

Impression and initial management plan

An initial clinical impression or differential diagnosis was recorded in 17 (11%) of admission notes. In contrast, a management plan was present in 113 (75%) of charts. While this indicates that treatment intent was often documented, the absence of a diagnosis or reasoning diminishes its clinical value. Importantly, none of the records (0 (0%)) included documentation of communication with the patient or their family regarding the diagnosis or plan.

Consent and confidentiality

Documentation regarding consent for history-taking and examination was present in 145 (97%) of charts, suggesting that clinicians were generally mindful of ethical obligations. Similarly, confidentiality statements were noted in 142 (95%), demonstrating good adherence to patient privacy standards.

## Discussion

Accurate clinical documentation on admission is fundamental to safe, effective patient care. The results of this audit demonstrate a striking deficiency in the completeness of admission notes in our pulmonology department, with only 1% of charts meeting all standards. This finding is notably worse than what has been reported in some other settings. For example, a recent audit of surgical inpatient records in Sudan found an overall compliance rate of about 59% with documentation standards, highlighting ongoing challenges like illegible entries, missing clinician identifiers, and inconsistent use of approved abbreviations [10]. In comparison, our audit’s overall compliance of 1% is alarmingly low, suggesting more severe or widespread documentation gaps in our setting. The UK surgical audit did note improvements in certain areas (such as better recording of date and time in later cycles) [11]. However, issues like unclear handwriting and absent doctor signatures persisted - issues we also observed (89% of our charts lacked clinician's name/signature, and many notes were hard to read). Thus, even though the contexts differ (surgical vs. medical admissions), the underlying problems of incomplete and unclear documentation appear to resonate across different healthcare environments.

Our findings echo those of other international audits that reported poor adherence to documentation protocols. In Basrah General Hospital (Iraq), for instance, 78% of inpatient records were judged to have generally poor documentation quality [1]. That audit particularly noted that history and examination sections were frequently incomplete, especially in surgical and medical wards. This parallels our observation that essential history elements (like history of presenting illness details, past history, and review of systems) were often missing or insufficient. Likewise, our audit reflects trends seen in a Sudanese teaching hospital’s audit, where only 41.1% of cases had the history of presenting complaint adequately documented and other critical sections (family, social history, etc.) had very low completion rates [12]. In our audit, only 25-35% of charts contained those same history details, aligning with the Sudanese experience of widespread omissions in clerking notes.

A similar audit of medical records in Pakistan (at a different institution) found that in 66% of cases the patient’s chief complaint was not recorded, and nearly half had no documented diagnosis [13]. We observed a comparable pattern: 25% of our charts lacked a clear presenting complaint, and an even larger proportion (89%) lacked a documented differential diagnosis or impression. This suggests that documentation habits in our region may generally be poor, possibly due to overwork, lack of training on medical record standards, or absence of accountability measures for documentation.

In examining specific documentation elements, our audit revealed many of the same deficiencies that others have noted. For instance, an audit of trauma patient records by Gupta et al. (2020) reported that only 10% of case notes contained a doctor’s signature, only 60% recorded the time of note entry, and nearly half lacked documented informed consent or important communication [14]. Our findings are consistent: only 11% of our notes identified the clinician, 28% had the entry time, and none recorded communication of the plan to the patient. These parallels reinforce that certain documentation lapses - such as omitting provider identification and timestamps - are common problems. They also highlight an area where focused improvements (like reminders or mandatory fields for signatures and timing) could yield quick benefits. Notably, poor legibility of notes was raised as an issue in the surgical audit by Singla et al. [11], which is a qualitative aspect we did not quantify but frequently observed in our review; illegible handwriting further diminishes the utility of a note, compounding the problem of missing content.

Our audit’s dismal results for medication and allergy documentation stand out as a major patient safety concern. Unfortunately, such issues are not unique to us. A clinical audit in a dental hospital setting in Lahore, Pakistan (using the CRABEL scoring system) similarly found very low rates of recording for key elements like diagnosis and drug allergies [13]. In our data, 0% of charts had allergy information. This is plainly dangerous and indicates an urgent need for intervention. The fact that some other audits in the region also found near-zero allergy documentation suggests that historically there has been insufficient emphasis on this critical item during admission clerking.

Encouragingly, there is evidence that targeted interventions can substantially improve documentation quality. Several studies have demonstrated improvements through the use of structured templates and proformas. Smallwood et al.’s multisite study showed that introducing standardized admission proforma forms significantly increased the completeness of documentation in medical admissions [2]. In our context, we believe a similar approach would be beneficial: a well-designed admission checklist or proforma for the pulmonology unit could prompt clinicians to fill in all necessary fields (including social and medication history which are currently often skipped).

A recent quality improvement project in Ethiopia by Bayisa et al. (2024) provides a striking example of what can be achieved. In that project, baseline documentation rates were extremely low - for instance, only 3.3% of records had fully clear initial documentation, and only 8.3% had legible numeric entries (such as dates) [15]. After implementing a multifaceted intervention (which included staff training, standardizing forms, and regular feedback), they reported improvements in those metrics to 70% and 82.8%, respectively. Moreover, the proportion of records with a proper date documented jumped from 62% at baseline to 95% post-intervention [15]. This dramatic enhancement underscores that poor documentation is a modifiable problem. By instituting structured documentation practices and engaging in continuous audit cycles, significant progress can be made even in a short time frame. The experience from Wallaga University Hospital aligns well with our needs - they used a “multidimensional quality improvement project” to address multiple points of failure, which likely included many of the same issues we identified (like signatures, legibility, completeness of histories).

Another instructive insight comes from a scoping review by Muinga et al. (2021), which examined how the design of paper-based records affects documentation quality [16]. The review found that involving end-users (the clinicians and nurses who actually fill the forms) in designing documentation tools is crucial. Features such as tick boxes, structured tables, and prompted fields (for example, a checkbox for “allergies: ___ None / Known ___”) can greatly improve completeness and accuracy. Muinga and colleagues noted that reducing reliance on free-text narrative (which tends to be inconsistently filled and often illegible) in favor of structured fields leads to better compliance and easier auditability [16]. Additionally, incorporating scoring systems like EWS charts into the admission paperwork can prompt staff to calculate and record those scores consistently. The implication for our setting is that a paper (or electronic) admission form tailored to pulmonology-perhaps with sections for smoking history, occupational exposure, etc., pre-listed-could ensure those items are not forgotten. Piloting such a form and iterating based on feedback, as recommended in the review, would likely be an effective strategy.

It is also important to recognize that documentation issues are not confined to inpatient hospital settings. Even in primary care, similar challenges exist. An audit of primary care medical records in Ireland found suboptimal documentation quality, with deficiencies in recording important patient information [17]. This broader perspective reinforces that documentation culture needs improvement across various levels of care, and lessons learned in one setting (like the importance of training and system support) are applicable to others.

Our audit clearly indicates that systemic factors underlie the documentation failures. It’s unlikely that all clinicians are simply forgetful; more plausibly, there may be high workload pressures, time constraints, or lack of awareness and training about the significance of thorough documentation. Additionally, the absence of a standardized template means each clinician writes notes in their own style, increasing the chance of omissions. There may also be a perception that certain details (like social history or medication lists) are not immediately relevant and can be skipped, an attitude that targeted education can change by highlighting the patient safety implications.

Limitations

This audit had some limitations worth noting. First, it was conducted at a single department in a single hospital, which may limit the generalizability of findings to other settings or specialties. However, the issues identified are common enough (and mirrored in external studies) that they likely reflect wider problems in documentation practices. Second, our data collection was retrospective and depended on the availability and legibility of notes; we may have under-counted an item if it was documented but not easily found (for example, medication lists scribbled in a corner could be missed). We tried to mitigate this by thoroughly double-checking each chart. Third, we focused on whether something was documented, not on the accuracy of what was written. A note might list a diagnosis that is incorrect, or a symptom description that is inaccurate. Such qualitative aspects were beyond our scope. Finally, while we used standardized criteria, the assessment of “complete vs incomplete” can carry some subjective judgment (e.g., how detailed does an HPI need to be to count as adequate). We addressed this by pre-defining objective markers (like the presence or absence of red-flag questions) and training the audit team for consistency. Despite these limitations, the audit provides a valid and stark measure of documentation quality in our department.

## Conclusions

This clinical audit revealed substantial gaps in the quality and completeness of admission documentation in the Pulmonology Department at MTI Mardan Medical Complex. While basic identifying information and patient consent were usually recorded, we found that essential components of the admission clerking, including detailed history of the presenting illness, past and social history, medication and allergy lists, thorough physical examination findings, and documented impression and plan, were frequently incomplete or entirely missing. Such documentation failures can compromise patient safety, hinder effective clinical decision-making, and disrupt continuity of care between providers. The audit’s findings align with reports from other hospitals nationally and internationally, indicating that inadequate documentation is a widespread issue that demands systematic remediation.

To address these deficiencies, we recommend a multifaceted quality improvement approach. First, a standardized admission template for the pulmonology unit should be introduced to prompt clinicians to document all required elements (history, exam, investigations, etc.). This structured format will reduce reliance on memory and ensure no critical information is overlooked. Second, targeted training sessions and workshops should be conducted to educate house staff on best practices in medical record keeping, emphasizing why each element (for example, medication history or smoking status) matters in patient care. Third, the department should institute regular internal audits or peer review of charts to provide ongoing feedback and sustain high standards. For instance, a quarterly audit cycle can track progress toward improvement goals (such as achieving >90% documentation of allergies and medications in all charts).

## Appendices

Audit tool: from history to plan - admission documentation in the Pulmonology Unit, MTI Mardan Medical Complex

A. Demographics and Basic Information

·       1. Patient's name, age, gender

·       2. Emergency contact number

·       3. Patient's MR Number

·       4. Date and time of history-taking

·       5. Name, grade, and signature of clerking doctor

·       6. PG initial assessment and arrival notes

B. Presenting Complaint (in Patient’s Own Words)

·       7. Presenting symptom in the patient’s own words

·       8. Duration of symptoms clearly mentioned

C. History of Presenting Complaint (HPC)

·       9. Description of the presenting symptoms

·       10. Symptom analysis: onset, location, duration, frequency, aggravating/relieving factors

·       11. Previous investigations or treatments

·       12. Presence or absence of red flag symptoms (e.g., hemoptysis, weight loss)

·       13. Functional impact on ADLs, sleep, or work

D. Past Medical History (PMH)

·       14. Relevant past medical/surgical history including respiratory and systemic illnesses

E. Family History

·       15. Relevant family and hereditary pulmonary history

F. Social History

·       16. Smoking history (current/past), pack-years

·       17. Type of smoking (e.g., cigarettes, shisha)

·       18. Alcohol or illicit drug use

·       19. Patient’s occupation and occupational hazards

·       20. Exposure to environmental pollutants or allergens

·       21. Living conditions (crowded housing/TB risk)

G. Medication History

·       22. Completeness of current medication list

·       23. Previous medications related to pulmonary conditions

·       24. Allergies and vaccination status (Flu, Pneumococcal, COVID)

H. Review of Systems (ROS)

·       25. Review of respiratory symptoms (e.g., cough, sputum, hemoptysis, dyspnea)?

·       25(A). Other systems briefly reviewed (e.g., cardiovascular, gastrointestinal, etc.)

I. Physical Examination

·       26. General appearance, respiratory rate, use of accessory muscles, chest deformities

·       27. Auscultation findings (wheezes, crackles, diminished breath sounds)

·       28. Vital signs noted (BP, HR, Temp, SpO2)

·       29. Early Warning Score (EWS/NEWS2) if applicable

J. Investigations

·       30. Relevant investigations ordered (CXR, CT, PFTs)

·       31. Laboratory results documented (e.g., ABG, CBC)

K. Impression and Plan

·       32. Differential diagnosis documented

·       33. Management plan outlined (medications, follow-ups, referrals)

·       34. Communication with patient/family about plan

L. Patient Consent and Confidentiality

·       35. Patient’s consent for history-taking documented

·       36. Confidentiality maintained throughout documentation

M. Overall Assessment

·       37. History documentation meets the standards set by the hospital or department
